# The reliability of the serial reaction time task: meta-analysis of test–retest correlations

**DOI:** 10.1098/rsos.221542

**Published:** 2023-07-19

**Authors:** Cátia M. Oliveira, Marianna E. Hayiou-Thomas, Lisa M. Henderson

**Affiliations:** Department of Psychology, University of York, York, North Yorkshire, UK

**Keywords:** reliability, meta-analysis, test–retest, serial reaction time task, individual differences

## Abstract

The Serial Reaction Time task, one of the most widely used tasks to index procedural memory, has been increasingly employed in individual differences research examining the role of procedural memory in language and other cognitive abilities. Yet, despite consistently producing robust procedural learning effects at the group level (i.e. faster responses to sequenced/probable trials versus random/improbable trials), these effects have recently been found to have poor reliability. In this meta-analysis (*N* = 7), comprising 719 participants (*M* = 20.81, s.d. = 7.13), we confirm this ‘reliability paradox’. The overall retest reliability of the robust procedural learning effect elicited by the SRTT was found to be well below acceptable psychometric standards (*r* < 0.40). However, split-half reliability within a session is better, with an overall estimate of 0.66. There were no significant effects of sampling (participants' age), methodology (e.g. number of trials, sequence type) and analytical decisions (whether all trials were included when computing the procedural learning scores; using different indexes of procedural learning). Thus, despite producing robust effects at the group-level, until we have a better understanding of the factors that improve the reliability of this task using the SRTT for individual differences research should be done with caution.

## 1. Introduction

The attempt to understand the role of individual differences in cognitive abilities has often led researchers to rely on well-established experimental tasks to capture effects of interest (e.g. [1,2]). However, such tasks were typically not designed to be sensitive at the individual level; in fact, in the examination of a group-level effect it can be desirable to reduce individual variability in order to better capture the phenomenon of interest. Consequently, an increasing number of well-established experimental tasks (e.g. Stroop task: [3]; Flanker task: B. A. [4]; Navon task: [5]) are being reported to have poor reliability [2,6]. This phenomenon is now referred to as the ‘reliability paradox’ [6]: specifically, tasks that produce robust group-level effects but fail to capture reliable individual differences, such that the rank order of participants' performance on the same measure lacks consistency and stability [6–8]. Reliability can refer to the ability of an instrument to consistently rank an individual's performance across time points (i.e. test–retest reliability or the *stability* of the test scores over different sessions) or subsets of the instrument's items within a session (i.e. split-half reliability) [8]. Low reliability in these experimental paradigms has often been attributed to the use of difference scores to isolate the effect of interest, because subtracting the experimental from the control conditions can reduce the variance between subjects [9]. Measurement error may also contribute to the poor stability of participant scores at test and retest where participants' scores will change non-systematically between sessions. Thus, if these tasks are mainly capturing measurement error, instead of reliable effects, individual difference studies based on these measures will fail to reflect real variation in individuals’ performance; and thus are also likely to produce attenuated correlations with other variables [1,6,9]. The opposite effect, whereby effect sizes are overestimated, may also occur by chance in small samples due to measurement error as demonstrated by Loken & Gelman [10]. Relatedly, and potentially partly a consequence of poor psychometric properties, experimental tasks that are thought to measure the same construct (e.g. Flanker and Stroop tasks) often fail to correlate with each other [1,11]. Thus, the ‘reliability paradox’ is an important problem to tackle if we are to advance our understanding of key cognitive constructs, particularly in the context of individual differences.

The SRTT is one such task, arguably the most widely used task to measure procedural learning and produce robust learning effects across settings, populations and task manipulations, in the face of poor psychometric properties [12–16]. In the SRTT [17], a stimulus appears on screen in one of four rectangles and participants are asked to respond as soon as possible to the position of the stimulus by pressing the corresponding key on the keyboard. Unbeknownst to the participants, the position of the stimulus follows a pattern of either deterministic or probabilistic nature. In the deterministic SRTT, blocks of patterned and random trials are alternated, with a larger number of patterned than random blocks. In probabilistic sequences, on the other hand, patterned trials are interspersed with random trials, with varying degrees of signal to noise ratio across tasks (a version of the probabilistic SRTT, known as ‘alternating SRTT', has the same number of sequenced and random trials). The nonverbal version of the SRTT is the most widely used, however the verbal SRTT has been more frequently adopted when working with Patients With Parkinson's Disease (e.g. [18,19]). Irrespective of which version of the task is used, procedural learning is proposed to be reflected by responses becoming faster for sequenced compared to random trials, as participants extract regularities from the sensory input and anticipate the position of the next stimulus.

Procedural memory is thought to underlie the acquisition and use of complex, sequence-based motor, perceptual and cognitive skills, including—according to an influential account—language [20,21]. This has led to interest in the role that procedural memory plays in driving individual variability in language development, whereby those with better procedural memory skills are expected to show better language proficiency and deficits in procedural memory can result in language and literacy impairments. However, despite the increasing interest in individual differences in procedural memory, researchers have used SRTTs in this context without much consideration for their psychometric properties [22]. Only more recently has the reliability of these tasks been questioned and found to be suboptimal (*r*s < 0.70; e.g. [13,14,16,23]).

It should also be noted that this finding of low reliability is mirrored in other measures thought to measure procedural learning that are not of focus here (e.g. the Hebb task: [15]; contextual cueing: [15]; artificial grammar learning: [24], word segmentation tasks: [13,24,25]). Indeed, Kalra *et al*. [12] found that a number of implicit learning tasks (i.e. SRTT, artificial grammar learning, probabilistic classification task), all of which are considered to index procedural learning showed below optimal test-retest reliability (*r*s < 0.45), and that inter-correlations among these measures were low, ranging from −0.18 to 0.32. In children, West *et al*. [23] observed poor test-retest reliability (*r* < 0.30) for verbal and nonverbal versions of the SRTT and Hebb tasks, and correlations between measures ranging from −0.18 to 0.24. While the small inter-correlations between procedural learning measures may be a consequence of the poor reliability of the individual tasks, it is also possible that these reflect a componential, rather than unitary, underlying construct [26].

Individual differences research is often used to better understand common latent processes across different tasks [11], however this endeavour is dependent on the psychometric properties of the measures adopted. Low correlations across tasks could reflect genuine underlying differences in the constructs being indexed, but they could also emerge as a consequence of attenuation due to low reliability. Thus, poor psychometric properties may limit theory building as the interpretation of available evidence is dependent on the reliability of the measures. If these tasks are capturing trial noise, instead of stable effects, inconsistencies between studies' conclusions may reflect situational variation.

Poor reliability may also contribute to the underspecification of cognitive constructs (e.g. inhibition:; attention: [2]). In relation to procedural memory, as noted by Bogaerts *et al*. [26], there is considerable vagueness in the demarcation between constructs that are thought to tap into rule-based learning (e.g. statistical learning, procedural learning, implicit learning); in the mapping between experimental tasks and these constructs; and, in how these constructs are related to language acquisition and difficulties. Therefore, a necessary step forward will be to establish the reliability of the SRTT and its impact on the interpretation of existing evidence. Thus, by examining the reliability of these tasks we can examine their capability to test procedural learning as a theoretical construct. A reliable procedural learning measure can also allow us to examine the relationship between procedural learning and other cognitive tasks which tap into the same or related processes and determine whether procedural memory represents a unitary construct, its demarcations, as well as its potential involvement in other cognitive abilities such as language and literacy acquisition (e.g. [12,13,23,25]).

Unsurprisingly then, the large body of research over the last decade that has used the SRTT to explore relationships between procedural memory and language and literacy abilities has produced inconsistent findings. Specifically, while some studies have shown that individuals with greater procedural learning ability also have better language and literacy skills (e.g. [27–29]), this finding is not ubiquitous (e.g. [13,23,30]). However, it is not clear whether differences in methods, reliability, or a combination of both, account for the inconsistent association between procedural learning and language. Namely, the studies which have examined the psychometric properties of the SRTT (e.g. [13,14,16,23]) vary on sample characteristics (e.g. age), design (e.g. interval between test and retest, use of the same or different SRTT sequences at different testing points), and the analytic method by which procedural learning is calculated (e.g. a simple difference between conditions, ratio scores, random slopes). However, the impact of these methodological variations on reliability is unknown. Thus, a systematic examination of the influence of such factors on reliability of the procedural learning effect is required, to better understand how to optimize the psychometric properties of the SRTT.

While many factors likely influence the test-retest reliability of the SRTT, here we focus on (i) sample characteristics (ii) task design, including the type of SRTT and the interval between test and retest, (iii) use of same or alternate forms of the SRTT and (iv) analytical decisions (e.g. index of learning effect (e.g. difference scores, whether to include all trials)). Regarding sample characteristics, there is some evidence that reliability of the SRTT is moderated by age, with lower test–retest reliability for children than adults [16,23]. One possibility is that lower retest reliability of procedural learning may be seen for children owing to age-related differences in the attentional and motivational demands of the SRTT [16]. There also appears to be a tendency for higher test-retest reliability for probabilistic (including both probabilistic and alternating SRTTs) than deterministic tasks as observed by Stark-Inbar *et al*. [14], where alternating sequences showed a test-retest reliability of 0.46 while for the deterministic SRTT the reliability coefficient was nearly zero (0.07). Furthermore, the study reporting the highest reliability thus far [16] used a probabilistic SRTT. The superior psychometric properties of probabilistic SRTT tend to be attributed to the lower likelihood of eliciting explicit awareness [14], since in deterministic SRTT the continuous repetition of the same elements of the sequence may contribute to its higher salience compared to sequences learnt in a noisier context [31]. In Stark-Inbar *et al*. [14], despite longer practice sessions in the alternating SRTT, participants who learnt the deterministic SRTT still demonstrated more evidence of explicit awareness. Furthermore, it is possible that procedural learning in probabilistic SRTTs is less confounded with fatigue, given that probabilistic sequences allow for the tracking of procedural learning throughout the task, instead of only in the last blocks as is common practice in deterministic sequences [32]. However, this is unlikely to be responsible for the differences in reliability between these variants of the SRTT, as [33] observed superior reliability when including only the last 3 blocks, instead of all trials.

While the effect of the interval length between test and retest has not yet been examined as a factor that influences reliability of the SRTT, evidence suggests that in cognitive and neuropsychological tests shorter intervals tend to lead to higher retest reliability coefficients than longer intervals [34]. This may be due to the possibility for true change in cognitive abilities to occur with longer intervals [35,34]. However, shorter intervals are also associated with greater opportunity for practice effects than longer intervals [36,37], where improvements across sessions may result from familiarity with the testing procedures, memory traces of the test items and the development of strategies [38]. The impact of practice effects on test–retest reliability may not be trivial, unless all participants show the same magnitude of improvement at retest, which is unlikely (e.g. [39,40]). Consequently such effects are likely to change the rank order of participants from test to retest. To reduce practice effects, researchers often administer alternate forms of a test; in the case of the SRTT, that can be achieved by using different sequences at test and retest, while the remaining task characteristics are kept consistent across sessions. However, alternate forms are often not sufficient to prevent practice effects from occurring [41]. This issue is relevant as practice effects in the SRTT were evident in Siegelman and Frost [13] where 64 out of 75 participants showed better performance at retest when using the same sequences at test and retest. However, its impact on reliability has rarely been experimentally tested. In [33], a positive effect of similarity between sequences at test and retest on the magnitude of procedural learning was observed, but not on test-retest reliability. Similar results were obtained by West and colleagues in two separate experiments, where reliability was assessed using the same (*r* = 0.21, [23]) or alternate forms of the SRTT (*r* = 0.26, [16]), thus suggesting that the adoption of alternate forms did not lead to significant changes in the coefficients. This suggests that even when steps are taken to avoid practice effects, performance may be susceptible to change across sessions; whether such changes are greater as a function of interval length between test and retest remains an open question.

Irrespective of task characteristics, a more recent debate has focussed on the methods used to capture reliability, whereby the ‘reliability paradox’ may not reflect a lack of stability in the underlying construct, but instead indicate that the use of point estimates to analyse reliability may fail to adequately model the data-generating process [42]. More concretely, instead of relying on point estimates, the suggestion is that methods for assessing reliability should integrate information at the individual and group level, while accounting for trial-by-trial variability [1,2,42]. Unfortunately, most evidence on the procedural learning effect and its reliability has used difference scores, with only a few studies which have controlled for overall speed by using ratio scores (e.g. [16,23]) or adopted random slopes [33,43,44]. Unlike the former indexes of procedural learning, random slopes fare better at integrating individual and group-level information and accounting for trial-by-trial variability. Thus, these model-based indexes may be able to capture better reliability, if the construct is indeed stable, as according to Stein's paradox [45] the best predictor of participants' true ability is not their own performance across sessions, but instead their adjusted performance that brings it closer to the observations of the group.

The aims of the present meta-analysis were threefold. First, we aimed to assess the frequency with which the reliability of the SRTT is reported. Second, we endeavoured to establish the test-retest reliability of the procedural learning effect as measured by the SRTT. While our preregistered objective was to examine test-retest reliability, the search strategy also produced studies examining split-half reliability, and thus this was also examined. Third, we aimed to examine which, if any, methodological factors influence the psychometric properties of this task (i.e. sample characteristics, task design including the interval between test and retest and use of same or alternate forms of the SRTT, analytical method for calculating procedural learning). With respect to methodological factors, we predicted that a) children would show poorer reliability than adults; b) longer intervals between test and retest would result in poorer reliability, c) that poorer reliability would be expected for difference scores than other measures of procedural learning (ratio scores and random slopes).

### 1.1. Study objectives

We present a meta-analysis of studies investigating the test–retest reliability of the SRTT. This investigation aims to assess the across-session stability of the SRTT, while considering possible moderating variables (e.g. age, length of interval between sessions). We predict that test–retest reliability will be suboptimal (*r* < 0.70) (H1), especially for children (H2) [16,23] and for longer intervals between test and retest (H3) as observed in other neuropsychological tasks (e.g. [37]). On the other hand, measures that take into account individuals’ speed (e.g. ratio, random slopes) are expected to have higher test–retest reliability than those which do not (e.g. difference scores) (H4) [16,33]. We also examine whether split-half reliability is closer to acceptable standards, as has been found in previous studies [16,23,46].

Exploratory analyses were conducted to determine further methodological characteristics that may influence test-retest reliability, namely the number of trials, use of the same or different sequences at test and retest, or SRTT variant (e.g. deterministic versus probabilistic sequence).

## 2. Methods

The protocol containing hypotheses, methods and analysis plan for this review was prospectively registered on the Open Science Framework (https://osf.io/uyqvt). All materials for this meta-analysis are available (https://osf.io/a65hn/), including the dataset and scripts necessary to replicate all reported analyses and plotting.

### 2.1. Search strategy

To ensure a comprehensive search strategy, a university librarian was consulted when developing the terms for each database. Literature was compiled by performing a full-text search in July 2021 on Medline, PsycINFO and Embase, as well as on BASE - Bielefeld Academic Search Engine for grey literature. Citation searching was also conducted to ensure that all relevant papers were identified. In order to ensure that the meta-analysis reflected the current state of the literature (as requested by reviewers), a second search was conducted in May 2023 by re-running the searches but limiting by publication date (2021–2023).

The following search string was used for Medline, PsycINFO and Embase: (Procedural learning OR Procedural memory OR Sequence learning OR Implicit learning OR Statistical learning OR Procedural knowledge.sh OR Serial Reaction Time task (.tw for PsycINFO)) AND (Reliab* OR Consistency OR Stab* OR Individual differences OR Valid* OR Psychometr* OR Measurement). For grey literature on BASE the following search string was used instead: (‘procedural learning’ ‘procedural memory’ ‘sequence learning’ ‘implicit learning’ ‘statistical learning’ ‘procedural knowledge’ ‘serial reaction time task’) AND (reliab* consisten* stab* ‘individual differences’ valid* psychometr* measurement*)

### 2.2. Selection of studies

One reviewer independently screened all articles and identified those relevant for the meta-analysis. This screening was done at the title and abstract level. At the full-article level the list of papers was screened by the first author to determine whether they fitted the inclusion criteria. To assess full-text eligibility the following inclusion criteria: i) Used a strictly visual deterministic, probabilistic or alternating SRTT with procedural learning computed as the difference between sequenced/probable and random/improbable trials; ii) Reported Pearson's correlation (or equivalent) coefficients between SRTT performance at two or more time points; iii) If the same results were published in multiple articles, these were only reported once in the meta-analysis; iv) Language of publication: English. Exclusion criteria were: i) Studies that used adaptations that considerably deviate from the task proposed by Nissen and Bullemer [17]; ii) Dual task paradigms; iii) Studies with active interventions or studying populations whose performance is expected to change over time (e.g. stroke patients).

In cases where it was unclear whether the manuscript met the inclusion criteria, a decision regarding its inclusion was reached by discussion between the three authors. Once the list of full articles was agreed upon, the first reviewer coded the data for the following items: a) Author/s; b) Publication year; c) Number of participants; d) Age of participants; e) Test-retest reliability; f) Split-half coefficient; g) Variant/version of SRTT (deterministic, probabilistic or alternating); h) Sequence complexity, i) Interval between sessions; j) Design: same or alternate version at 2nd test-point, k) Total number of trials completed, l) Number of trials included when computing the reliability measure: all trials or only last blocks, m) index of procedural learning (difference scores, ratio scores or random slopes—ratio and random slopes were collapsed due to the small number of studies examining measures other than difference scores).

**Figure 1.**
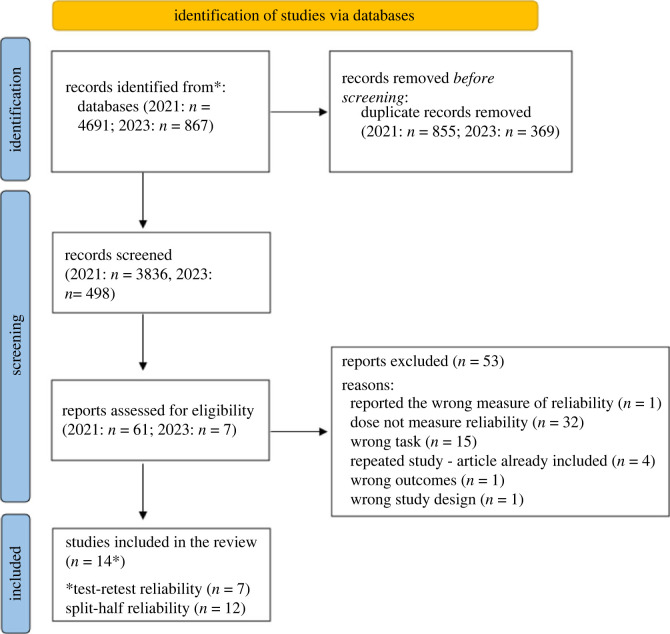
PRISMA flowchart showing selection of studies for meta-analysis on the reliability of the SRTT.

The PRISMA flow diagram [47] in figure 1 presents the number of records identified, included, and excluded; as well as the reasons for exclusions.

### 2.3. Statistical analyses

The analyses were carried out using R (v. 4.1.1) [48]. All continuous moderators were mean centred. Centering moderators does not change the random coefficients. All correlation coefficients were converted from Pearson's r to Fisher's z scale as Pearson's r is not normally distributed [45].

The *metafor* package [49] was used for model fitting. Random effects working models were fitted following the guidelines from Pustejovsky & Tipton [50] for model specification to reflect the levels of dependency in the dataset. Since research groups contributed multiple correlation coefficients from the same samples, to deal with the lack of independence across effect sizes and avoid reducing power by calculating the average effect sizes for these studies, multilevel models were estimated using the function *rma.mv()* from the *metafor* package [49]. As recommended by Pustejovsky & Tipton [50], robust variance estimation standard errors, hypothesis tests and confidence intervals for the meta-regression coefficient estimates were computed using the functions *coef_test()* or *conf_int()* from the *clubSandwich* [51] to guard against model misspecification [50]. Since these multilevel models represent working models which may fail to fully represent the dependence structure, robust variance estimation methods were used as these do not require exact knowledge of the dependence, thus even if the working model is misspecified, the estimates will be unbiased [50]. The correlation of the sampling errors within clusters (rho) was set at 0.80, and sensitivity analyses were conducted to determine whether this decision impacted the overall estimate.

An intercept-only model was fitted to estimate the overall test-retest reliability (i.e. the average correlation coefficient between test and retest) of the SRTT. Following the intercept-only model, separate meta-regression models were performed for each mediator variable (e.g. age, total number of trials) to determine whether any of these factors influence the test-retest reliability of the SRTT. After performing the meta-analytic calculations, Fisher's *z* effect sizes were converted back to Pearson's *r* for reporting the average correlation and 95% CI for each model. We first started by fitting a reduced model which included only one effect size per sample. When multiple reliability estimates were available for the same sample, difference scores were adopted as a default measure unless such measure was not available, as these better represent current practices in the field of procedural learning. Finally, a second model was fitted (full model) which includes all effect sizes, thus allowing for direct comparisons between analytical decisions across studies.

### 2.4. Bias analysis

Study heterogeneity was analysed using the Q-test for heterogeneity [52] which reflects the ratio of observed variation to within-study variance. Cook's distances and studentized deleted residuals (or externally studentized residuals) were used to identify potential influential and outlier cases, respectively, as these may distort the conclusions of the meta-analysis [53]. Cook's distances provide information about the leverage of each effect size by excluding each study in turn and determining its impact on the overall estimate. Studentized deleted residuals, on the other hand, were used to identify potential outlier points, i.e. absolute studentized deleted residuals larger than 1.96 [53].

To detect evidence of bias, funnel plots, contour-enhanced funnel plots, as well as Egger's regression test [54], were used to check for funnel plot asymmetry. Contour-enhanced funnel plots are an extension of funnel plots, as the areas of statistical significance have been overlaid on the funnel plot. By adding these contours, it is possible to determine whether there are potential missing studies in areas of no significance, thus suggesting that the asymmetry may be due to publication bias [55]. Following the recommendations by Sterne *et al*. [56], these were interpreted with caution given the small number of studies (<10 studies) included in the present meta-analysis.

Finally, given that our group [33,57,58] has comprehensively examined the reliability of the SRTT across settings (in laboratory and online), with various measures (e.g. difference scores and random slopes) and using different levels of similarity between sequences, follow-up models were fitted to the data after excluding the effect sizes from our group.

## 3. Results

In total, the meta-analysis includes 7 independent studies [12–14,16,23,33,57–59] (citations marked with a dagger) summarizing 36 effect sizes and data from 719 participants (*M* = 20.81, s.d. = 7.13), comprising 199 children and 520 adults. Thus, it was observed that, despite the frequent adoption of the SRTT to analyse procedural memory (as of September 2021 a Google Scholar search of ‘Serial Reaction Time task’ yields 13 300 results), only a small fraction of studies reported a test-retest reliability estimate. All studies were published between 2015 and 2023.

### 3.1. Test–retest reliability

**Figure 2.**
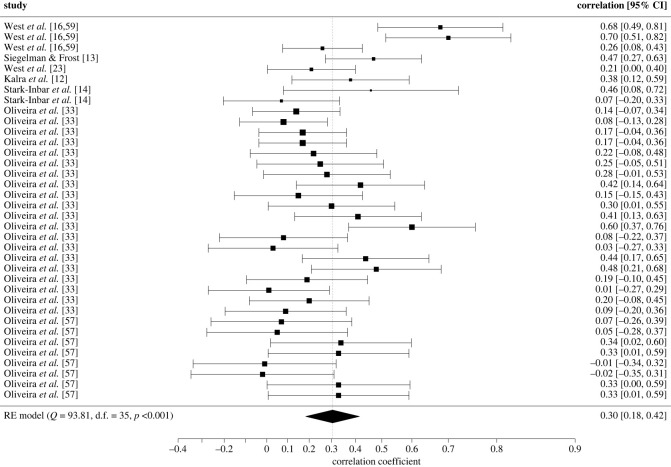
Forest plot showing the observed outcomes and the estimate of the multilevel model for test-retest reliability.

A multilevel mixed effects model was fitted to the test–retest reliability data. In this first model, only a single reliability score was included per experiment, with difference scores being chosen when more measures were available. Only the study by Kalra *et al*. [12] did not report difference scores, reporting instead ratio scores. This (reduced, i.e. only includes one effect size per experiment) model revealed a significant and suboptimal pooled test–retest reliability across studies and measures (Fisher's *z* = 0.29, 95% CI [0.16, 0.43], s.e. = 0.07, *z* = 4.31, *p* < 0.001), with an equivalent test–retest reliability of *r* = 0.28, 95% CI = [0.16, 0.40]. Follow-up RVE estimates were computed to guard against model misspecification and correct for the small sample size; these yielded results consistent with the multilevel model (s.e. = 0.07, *t*_5.04_ = 4.23, *p* = 0.008). A forest plot showing the observed outcomes and the estimate based on the random-effects model is shown in figure 2.

Because the studies by [33,57,58]) contributed a relatively large amount of data (5 effects and a total of *N* = 306 participants) to the meta-analysis, a second multilevel model was fitted after removal of the effect sizes from this laboratory to determine their impact on overall reliability. Suboptimal test-retest reliability was still observed, with only a small increase in the estimated reliability (Fisher's *z* = 0.38, 95% CI [0.21, 0.56], s.e. = 0.09, *z* = 4.21, *p* < 0.001), which corresponds to *r* = 0.37, 95% CI = [0.20, 0.51].

According to the results of the Q-test, there is considerable heterogeneity in the estimation of the test-retest reliability of the SRTT (*Q*(df = 11) = 26.51, *p* = 0.005). Thus, this variability across studies was explored through meta-regressions. To achieve this, a second (full) model including all effect sizes was fitted to determine the impact of some frequent methodological decisions. This model revealed a similar average test-retest reliability (Fisher's *z* = 0.31, 95% CI [0.19, 0.44], s.e. = 0.07, *z* = 4.80, *p* < 0.001), corresponding to a Pearson's correlation of 0.30, 95% CI = [0.18, 0.42]. As before, a follow-up model was fitted which excluded the findings from [33]; this again yielded a small increase in the reliability coefficient, which was however still far from optimal (Fisher's *z* = 0.39, 95% CI [0.20, 0.58], s.e. = 0.10, *z* = 4.08, *p* < 0.001; *r* = 0.37, 95% CI [0.20, 0.52]).

Given the clustered nature of the models presented, influential and outlier effect sizes were identified at the various levels. At the study and experimental level, the study conducted by West, Shanks *et al*. [16] in adults was identified as an influential point which upwardly biased the overall estimate. In the opposite direction, [33] was identified as an influential point at the study level for both models, while the experiment conducted by West, Shanks *et al*. [16] in children was influential only at the experimental level. The effect sizes from West, Shanks *et al*. [16] were identified as outliers for both models (reduced: only adult data; full model: both child and adult effect sizes).

Moderator analyses revealed no evidence of a significant moderating effect of age, total number of trials, or test–retest interval on the magnitude of the test-retest reliability coefficient (*ps* > 0.05). For categorical variables (i.e. measure, type of SRTT, ISI, trials included when computing the reliability measure, SRTT version), the test–retest reliability was significantly different from zero for at least one level of the moderator variable. However, given the small sample size, only RVE estimates will be interpreted. These revealed an average test-retest reliability that was significantly different from zero across measures, irrespective of which measure was used. A numerical but non-significant advantage was observed for ratio and random slopes compared to difference scores (*F*_1,1.46_ = 2.57, *p* = 0.293). A slight numerical, but not significant, advantage was also observed for SRTTs with an interstimulus interval versus those without (*F*_1, 2.85_ = 0.266, *p* = 0.644), as well as for studies which computed procedural learning using the last blocks of the experiment rather than for the whole task (*F*_1,1.38_ = 0.86, *p* = 0.487). Probabilistic SRTTs also yield slightly better test-retest reliability than deterministic tasks, but again this difference did not reach significance (*F*_1, 1.52_ = 0.247, *p* = 0.682).

### 3.2. Publication bias

**Figure 3.**
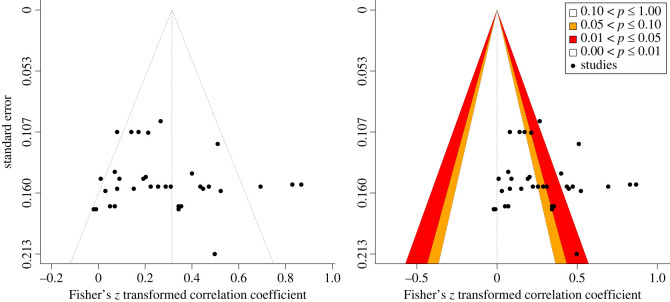
Funnel plot showing effect sizes plotted against standard error for test-retest reliability. (*a*) funnel plot (left panel) and (*b*) contour-enhanced funnel plot (right panel).

Visual inspection of the funnel and contour plots shown in figure 3 for all effect sizes does not reveal evidence of plot asymmetry or overrepresentation of studies in the significance contours, which is consistent with the non-significant Egger's test with standard error as a predictor (*b* = 3.23, *p* = 0.075). Thus, there is no evidence of publication bias.

### 3.3. Split-half reliability

While the search strategy was aimed towards identifying studies which examined the test-retest reliability of the SRT, the search results also yielded 12 studies reporting split-half reliability comprising 1605 participants (*M*_age_ = 19.49, s.d. = 7.92); an exploratory analysis examining this was carried out [16,23,33,43,44,57,58,60–65]

As for test-retest reliability, when studies computed split-half reliability using multiple indexes, only difference scores were selected for this first model as this index is the most commonly used in the field. This was only the case for the studies by [33]. The overall split-half reliability of the SRTT was higher than its test-retest reliability, with a pooled effect size of *r* = 0.63, 95% CI [0.52, 0.72] (Fisher's *z* = 0.74, 95% CI [0.58, 0.90], s.e. = 0.08, *z* = 9.11, *p* < 0.001); this was unaffected when using RVE (s.e. = 0.08, *t*_6.78_ = 9.83, *p* < 0.001). Sensitivity analyses revealed that the estimates were robust to distinct values of rho with the estimates ranging from 0.740 to 0.741. When removing the effect sizes from [33] there was a negligible improvement in the split-half estimate *r* = 0.65, 95% CI [0.49, 0.77] (Fisher's *z* = 0.77, 95% CI [0.54, 0.1.01], s.e. = 0.12, *z* = 6.37, *p* < 0.001).

Following the high degree of heterogeneity in the estimates of split-half reliability in the reduced model (only one effect size per experiment) (*Q*_25_ = 215.84, *p* < 0.001), a full model with all effect sizes was performed to explore whether any of the sampling and/or methodological factors impact the split-half reliability of the SRTT.

**Figure 4.**
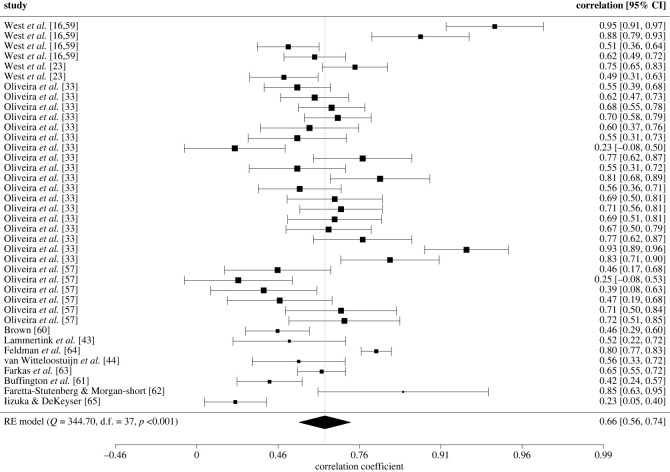
Forest plot showing the observed outcomes and the estimate of the multilevel model for split-half reliability

When all effect sizes were included there was a slight increase in the split-half reliability, *r* = 0.66, 95% CI [0.56, 0.74] (Fisher's *z* = 0.79, 95% CI [0.63, 0.95], s.e. = 0.08, *z* = 9.71, *p* < 0.001; RVE (s.e. = 0.07, *t*_5.80_ = 10.80, *p* < 0.001). Removal of the effect sizes by [33,57,58] did not change the findings, *r* = 0.65, 95% CI [0.49, 0.77] (Fisher's *z* = 0.77, 95% CI [0.53, 1.01], s.e. = 0.12, *z* = 6.37, *p* < 0.001; RVE: s.e. = 0.11, *t*_7.79_ = 6.81, *p* < 0.001). The study by West, Shanks *et al*. [16] with adults was identified as an influential case at all levels upwardly biassing the estimate for both the full and reduced models. Additionally, at the effect size level, the study by Iizuka & DeKeyser [65] was also identified as a potential influential point, negatively biassing the overall reliability estimate. Finally, the effect size from West, Shanks *et al*. [16] with adults and the highest effect size from [33] were identified as outliers. A forest plot showing the observed outcomes and the estimate based on the multilevel model is shown in figure 4 and tables 1, 3 and 4.

**Table 1.** Overview of the study sample characteristics for each individual experiment in our test-retest reliability meta-analysis.

exp	study	age (years)	sequence complexity	SRTT type	trials	interval (days)	design	ISI (ms)	testing setting
*1*	West *et al*. [16,59]	*25.33*	*SOC*	*probabilistic*	*3000*	*2 to 3*	*different*	*250*	*in-person*
*2*	West *et al*. [16,59]	*7.68*	*SOC*	*probabilistic*	*2000*		*different*	*250*	*in-person*
*3*	Siegelman & Frost [13]	*24.1*	*SOC*	*probabilistic*	*1920*	∼ *3 months*	*same*	*0*	*in-person*
*4*	West *et al*. [23]	*8.08*	*SOC*	*probabilistic*	*1000*		*same*	*250*	*in-person*
*5*	Kalra *et al*. [12]	*17.69*	*SOC*	*deterministic*	*1536*	*13.89*	*different*	*250*	*in-person*
*6*	Stark-Inbar *et al*. [14]	*21.20*	*SOC*	*alternating*	*7650*	*2 to 5*	*different*	*120*	*in-person*
*7*	Stark-Inbar *et al*. [14]	*21.20*	*SOC*	*deterministic*	*2520*	*0 to 5*	*different*	*100*	*in-person*
*8*	Oliveira *et al*. [33]	*19.18*	*SOC*	*probabilistic*	*2000*	*7*	*different*	*0*	*in-person*
*9*	Oliveira *et al*. [33]	*20.09*	*SOC*	*probabilistic*	*2000*	*7*	*different*	*0*	*in-person*
*10*	Oliveira *et al*. [33]	*28.52*	*SOC*	*probabilistic*	*2000*	*7.85*	*different*	*0*	*online*
*11*	Oliveira *et al*. [33]	*30.41*	*SOC*	*probabilistic*	*2000*	*7.85*	*different*	*250*	*online*
*12*	Oliveira *et al*. [57]	*26.20*	*SOC*	*probabilistic*	*2000*	*7.76*	*different*	*0*	*online*

Meta-regressions revealed results consistent with the findings for test-retest reliability (table 2). There was no evidence that age and the total number of trials had a moderating effect on split-half reliability (*p*s > 0.05). For categorical variables, there was no statistical difference between any of the contrasts, although random slopes showed a numerically slightly higher split-half reliability compared to difference scores (*F*_1, 1.14_ = 1.10, *p* = 0.439) and there appeared to be a slight numerical advantage of having an interstimulus interval (250 ms), (*F*_1, 5.11_ = 0.573, *p* = 0.482). No difference was observed in the split-half reliability between types of SRTTs (probabilistic versus alternating: *F*_1, 2.74_ = 0.002, *p* = 0.968; deterministic versus alternating: *F*_1, 3.91_ = 0.0007, *p* = 0.968; probabilistic versus deterministic: *F*_1, 2.85_ = 0.0002, *p* = 0.990).

**Table 2.** Results of all separate meta-regressions with moderator variables for test-retest reliability. *Note*. s = number of studies; exp = number of experiments; ES = number of effect size estimates; z’ = Fisher's z values; r = Pearson's R correlation; standard errors (SE) and *z* values for individual levels of a moderator; *p* values correspond to z or t - values; 95% CI corresponds to the Fisher's z.

moderator (bolded) and levels	S	exp	ES	test of moderators		meta regression							RVE			
				QM	*p*	Fisher's z	*r*	s.e.	*z*	*p*	95% CI		s.e.	*t*	d.f.	*p*
**age**	7	12	36	0.59	0.443	0.04	0.04	0.05	0.77	0.443	−0.06	0.13	0.05	0.72	2.46	0.536
**measure**	7	12	36	27.92	<0.001	__	__	__	__	__	__	__	__	__	__	__
difference scores	6	11	20	__	__	0.28	0.27	0.07	3.92	<0.001	0.14	0.42	0.08	3.33	4.73	0.023
ratio/random slopes	4	7	16	__	__	0.40	0.38	0.08	5.28	<0.001	0.25	0.55	0.07	5.74	3.67	0.006
**# of trials**	7	12	36	1.72	0.190	0.06	0.06	0.05	1.31	0.190	−0.03	0.15	0.08	0.80	1.25	0.548
**type of SRTT**	7	11	35	15.01	<0.001	__	__	__	__	__	__	__	__	__	__	__
deterministic	2	2	2	__	__	0.23	0.23	0.18	1.29	0.197	−0.12	0.59	0.17	1.42	1.00	0.390
probabilistic	5	9	33	__	__	0.33	0.32	0.09	3.65	<0.001	0.15	0.50	0.09	3.73	3.43	0.027
**ISI**^a^	6	10	34	18.85	<0.001	__	__	__	__	__	__	__	__	__	__	__
0	3	5	25	__	__	0.30	0.29	0.10	2.93	0.003	0.10	0.50	0.06	5.28	1.59	0.054
250	4	5	9	__	__	0.36	0.35	0.10	3.39	<0.001	0.15	0.56	0.12	2.88	2.71	0.072
**trials included**	7	12	36	22.45	<0.001	__	__	__	__	__	__	__	__	__	__	__
all trials	5	10	18	__	__	0.30	0.29	0.07	4.09	<0.001	0.15	0.44	0.07	4.26	3.82	0.015
last blocks	4	7	18	__	__	0.35	0.34	0.08	4.55	<0.001	0.20	0.50	0.08	4.25	3.33	0.019
**version of sequence at retest**	7	12	36	21.09	<0.001	__	__	__	__	__	__	__	__	__	__	__
same	2	2	2	__	__	0.36	0.35	0.16	2.21	0.027	0.04	0.68	0.15	2.42	1.00	0.250
different	5	10	34	__	__	0.31	0.30	0.08	4.03	<0.001	0.16	0.46	0.08	3.94	2.97	0.030
**Interval between test & retest^b^**	3	6	29	0.39	0.531	0.03	0.03	0.04	0.63	0.531	−0.06	0.11	0.03	0.57	1.17	0.659

^a^As only one effect size was available for ISIs of 100 and 120, both from Stark-Inbar *et al.* [14], these were not included in the analysis/

^b^Only a small number of experiments (*n* = 6; [12,33,57,58]) reported the mean interval between sessions.

**Table 3.** Overview of the study sample characteristics for each individual experiment in our split-half reliability meta-analysis.

exp	study	age (years)	sequence complexity	SRTT type	number of trials	ISI (ms)	testing setting
1	West *et al*. [16,59]	25.333	SOC	probabilistic	1500	250	in person
2	West *et al*. [16,59]	7.68	SOC	probabilistic	1000	250	in person
3	West *et al*. [23]	8.08	SOC	probabilistic	500	250	in person
4	Oliveira *et al*. [33]	19.18	SOC	probabilistic	1000	0	in person
5	Oliveira *et al*. [33]	20.09	SOC	probabilistic	1000	0	in person
6	Oliveira *et al*. [33]	30.41	SOC	probabilistic	1000	250	online
7	Oliveira *et al*. [33]	28.52	SOC	probabilistic	1000	0	online
8	Oliveira *et al*. [57]	26.20	SOC	probabilistic	1000	0	online
9	Brown [60]	31.97	SOC	probabilistic	1008	0	in person
10	Lammertink *et al*. [43]	9.083333	FOC	deterministic	380	250	in person
11	Feldman *et al*. [64]	14.4	FOC	deterministic	508	500	in person
12	van Witteloostuijn *et al*. [44]	9.75	FOC	deterministic	380	250	in person
13	Farkas *et al*. [63]	21.61	SOC	alternating	3825	0	in person
14	Buffington *et al*. [61]^1^	19.30	SOC	alternating	1700	0	in person
15	Faretta-Stutenberg & Morgan-Short [62]	20.10	SOC	alternating		0	in person
16	Iizuka & DeKeyser [65]	20.06	SOC	probabilistic	1009	0	in person

**Table 4.** Results of all separate meta-regressions with moderator variables for split-half reliability. *Note*. s = number of studies; exp = number of experiments; ES = number of effect size estimates; z’ = Fisher's z values; r = Pearson's R correlation; standard errors (SE) and z values for individual levels of a moderator; p values correspond to z or t - values; 95% CI corresponds to the Fisher's z.

moderator (bolded) and levels	s	Exp	ES	test of moderators		meta regression							RVE			
				QM	p	Fisher's z	r	SE	z	p	95% CI		SE	t	df	P
**age**	12	16	38	0.65	0.420	0.06	0.06	0.08	0.81	0.420	−0.09	0.21	0.06	1.01	5.01	0.359
**measure**	12	16	38	88.38	<0.001	__	__	__	__	__	__	__	__	__	__	__
difference scores	10	14	24	__	__	0.73	0.62	0.09	8.18	<0.001	0.56	0.91	0.09	8.54	6.13	<0.001
ratio/random slopes	4	7	14	__	__	0.92	0.73	0.11	8.50	<0.001	0.70	1.13	0.16	5.78	2.70	0.014
**total # of trials**	11	15	37	0.06	0.80	0.02	0.02	0.06	0.25	0.802	−0.10	0.14	0.04	0.383	1.59	0.746
**type of srtt**	12	16	38	82.29	<0.001	__	__	__	__	__	__	__	__	__	__	__
deterministic	3	3	3	__	__	0.79	0.66	0.22	3.54	<0.001	0.35	1.22	0.18	4.38	1.99	0.049
probabilistic	6	10	32	__	__	0.79	0.66	0.10	7.62	<0.001	0.59	0.99	0.10	8.19	2.98	0.004
alternating	3	3	3	__	__	0.78	0.65	0.23	3.42	<0.001	0.33	1.23	0.21	3.70	1.93	0.07
**ISI^a^**	11	15	37	81.11	<0.001	__	__	__	__	__	__	__	__	__	__	__
0 ms	7	9	25	__	__	0.72	0.62	0.11	6.48	<0.001	0.50	0.93	0.12	6.20	3.54	0.005
250 ms	5	6	12	__	__	0.85	0.69	0.14	6.25	<0.001	0.58	1.11	0.12	6.86	3.33	0.005

^a^Only the study conducted by Feldman *et al.* [64] included an ISI of 500 ms, therefore it was not included in this analysis.

### 3.4. Publication bias

**Figure 5.**
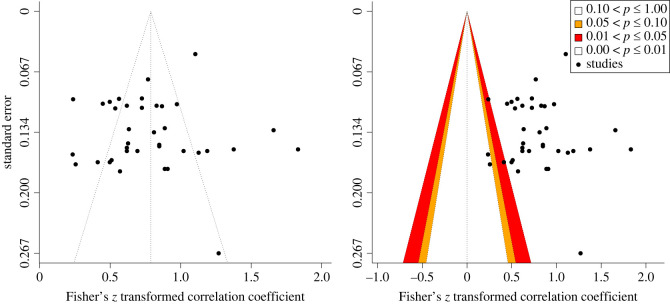
Funnel plot showing effect sizes plotted against standard error for split-half reliability. (*a*) funnel plot (left panel) and (*b*) contour-enhanced funnel plot (right panel)

Visual inspection of the funnel and contour plots (figure 5) shows no clear evidence of plot asymmetry or concentration of the effect sizes in the significance contours. This pattern is consistent with the non-significant Egger's test (*b* = 1.77, *p* = 0.339). Thus, there is no evidence of publication bias.

## 4. Discussion

The reliability of the SRTT is clearly understudied as, despite being one of the most commonly used experimental paradigms in procedural memory research, only 7 studies have reported the psychometric properties of this task. Drawing on these studies, as expected (H1), the present meta-analysis provides evidence that the SRTT generally does not meet the standards for adequate test–retest reliability (i.e. *r* > 0.70, [8,67]), with an average test–retest reliability coefficient of between 0.28 and 0.30. This low test-retest reliability was observed irrespective of sampling and methodological considerations, which will be further discussed below. Split-half reliability, on the other hand, was better, with reliability coefficients varying between 0.63 and 0.66. Thus, this meta-analysis confirms poor across-session reliability for procedural learning, in the context of near-acceptable within-session reliability as previously observed by [33].

For test-retest reliability, no single sampling, methodological or analytical decision examined here appeared to be sufficient for reaching the threshold of adequate retest reliability. While we recognize that other task and sample characteristics beyond the ones investigated in this meta-analysis might be of relevance, this meta-analysis only included the most frequently adopted design of the SRTT (i.e. visual SRTT with four locations) in typically developing samples. To our knowledge, only West *et al*. [23] examined the reliability of a verbal version of the SRTT (split-half reliability: 0.17–0.27, test-retest reliability: −0.001). Although there were small numerical improvements in reliability for indexes of procedural learning that account for participants' speed (i.e. ratio and random slopes) as opposed to difference scores, and for the probabilistic version of the SRTT when compared to deterministic tasks, neither of these factors significantly influenced reliability. For split-half reliability, numerically (but not significantly) better reliability was also observed for random slopes over difference scores and for studies with an interstimulus interval (250 ms) when compared to those without (0 ms). Counter to our predictions, we found no evidence of an effect of age and length of the test–retest interval on reliability. Yet, when considering the sample size, it is possible that the absence of a moderating effect of these factors may reflect a lack of power since [23], and [16] reported a clear pattern of better test-retest and split-half reliability in adults than children. Furthermore, when we adopt a dichotomous approach (children versus adults) when examining the test-retest reliability, the overall reliability is only significant for adults (*r* = 0.36) and not children (0.11), but there is still no significant difference between age groups. Additionally, the variability in the time scale between test and retest in this sample was quite limited. Finally, the absence of a moderating effect of the total number of trials may be due to the fact that all studies included in this meta-analysis used 380 or more trials per session. Even though we found no evidence for an effect of the number of trials, this should not be interpreted to suggest that the number of trials does not impact the reliability of the SRTT, primarily because experimental studies focusing on group-level effects often adopt a considerably smaller number of trials than the individual-differences studies reported here (e.g. as low as 192 trials in group-level studies, [68] compared to 380–3825 in individual-differences studies using the SRTT) [29,68–70] It is possible that increasing the number of trials even further, beyond the range of 380–3825, could lead to improvements in test-retest reliability by reducing trial noise [11]. However, based on recent findings from Farkas *et al*. [63], only small gains in reliability were observed after 20 blocks (1700 trials) with the alternating SRTT.

Although the SRTT is well known for producing a robust procedural learning effect at group-level, the findings from the present study raise questions about its suitability for individual differences research, since poor reliability contributes to attenuation of the association between measures [1]. Hierarchical modelling has been suggested as a way to disattenuate correlations [1,71], however, despite producing less biased estimates than naive sample-effect correlations, the estimates are still highly variable [1]. Thus, further investigation into the reasons for the lack of retest reliability is warranted, alongside efforts to develop tasks that are more suitable for eliciting adequate and reliable between subject variability. More reliable measures of procedural learning will help clarify whether the absence of correlations between procedural learning in the SRTT and language and literacy [43,59] and between different measures of procedural learning [12,13,25,59] reflect a real lack of shared variance between these measures or whether individual differences fail to be captured due to poor reliability. Thus, resolving the reliability issue is not only of statistical importance, but will also help to clarify theoretical issues pertaining to procedural learning as a construct and its role in language and literacy development and disorders.

Despite the common attribution of poor reliability to the use of difference scores [72,73], poor test-retest reliability cannot be solely explained by the metrics used to index procedural learning, in light of similarly poor retest reliability when ratio scores or random slopes are used, and adequate split-half reliability regardless of the index used. Instead, it may be necessary for the field to adopt indexes that more closely resemble the data generating process and that account for processing speed and trial noise (e.g. through Bayesian hierarchical modelling, see [42,74]), as these approaches are likely to fare better at capturing the construct of interest [2,42]. Unlike difference scores, which only provide point estimates of the individuals’ performance, hierarchical models include information at the group and individual level, which has been found to better capture individuals' true ability [42]. In the present meta-analysis we examined ratio and random slopes as these represent current practices in the field, as expected, model-based parameters appear to fare better at capturing the reliability of the SRTT task however future research using more sophisticated models may be better able to separate measurement error from true individual differences.

In addition to resolving measurement and analytical challenges, it may be fruitful for future research to consider how performance in the SRTT may interact with other cognitive processes. For example, procedural learning effects have been shown to be positively associated with attention, with individuals with better sustained attention skills showing a larger procedural learning effect [16,33,75]. Thus, if individuals’ alertness and motivation were to change between test and retest that would be likely to manifest in variations in their performance, consequently affecting the consistency of their ranking between test and retest. This may be expected to be more marked in children, given that their attentional skills are still developing [76] and attentional fluctuations have been previously found to decrease between childhood into young adulthood [77,78]. These changes between test and retest would be less influential for split-half reliability, as they would represent shorter-scaled differences in performance that would be captured in both odd and even trials; this is consistent with the finding of better split-half than retest reliability in the SRTT.

Crucially, individual differences research assumes that there are stable differences between individuals in the construct of interest which may influence individuals' accumulated experience and learning over the long term, and which, if adequately captured, would likely be reflected in high levels of stability over time. However, it is possible that the poor reliability of the procedural learning effect does not reflect a problem with the paradigm. Instead, it may indicate that procedural learning itself does not vary greatly in the general population;, it may be that a minimum level of procedural learning ability is sufficient for the acquisition of cognitive and motor skills and habits. Therefore, the magnitude of the difference scores may carry only limited meaning, and it may be more important whether the individual is able to extract any knowledge from the task, irrespective of its magnitude. This is in line with A. S. Reber's [79] proposal that procedural learning, due to being evolutionarily old, is expected to show little between subject-variability, unlike declarative memory. Following from this, if individuals do not differ enough from one another then measurement fluctuations will lead to substantial changes in ranking order.

Alternatively, it is likely that the SRTT is not a process-pure measure of procedural learning, but that both procedural and declarative memory systems are involved when performing the SRTT [80]. Therefore, it is possible that as individuals develop explicit knowledge of the sequence, the strategies they adopt might change. Explicit awareness of the sequence is not inherently problematic for the stability of the procedural learning effect, as long as its impact is similar across participants, thus preserving the rank order between test and retest. However, this is unlikely to be the case given that the emergence of explicit knowledge has been shown to vary depending on participants' characteristics such as age [81] and sleep architecture [82]. Probabilistic sequences are often considered purer measures of implicit procedural learning as they tend to yield less explicit awareness of the underlying sequences than deterministic sequences [83]. If poorer reliability was associated with more explicit awareness, we would potentially expect better reliability for the probabilistic SRTTs, however that is not supported by our findings. Nonetheless, more controlled designs which directly analyse the impact of explicit awareness on the reliability of the SRTT are required.

Similarly, practice effects, which are often observed in memory tasks [36,41,84,85], would also be expected to affect test–retest reliability more than split-half reliability. In the context of procedural memory, there is also the question of the extent to which task stability should be expected: individual performance is expected to change with practice, with an initial stage of procedural learning usually being marked by improvements in speed and accuracy, followed by consolidation and later automatization of the learnt probabilistic information [86,87]. If these stages are captured by the SRTT, at least until automatization has occurred, then performance should be expected to change across time. Further, the ranking between participants may also change as a result of individual differences in the rate at which participants make the transitions between stages of learning (as has been observed in other memory tasks [88,89]). For a discussion of practice effects in the context of the alternative SRTT see Farkas *et al*. [63].

To summarize, the usefulness of the SRTT as a measure of individual differences in procedural learning relies on the stability of performance at test and retest. The lack of reliability demonstrated in this meta-analysis may reflect a lack of sensitivity, which could in principle be overcome by further refinements of the task, which might include different response types: for example, it may be that oculomotor versions of the SRTT (which were not included in the current study) are more sensitive to stable individual differences than the classic motor version of the task. On the other hand, it may be that even with such refinements, reliability will not improve because the SRTT relies too heavily on other constructs, such as attention [33], which are likely to vary across sessions. An alternative possibility is that the SRTT is in fact stable, but the statistical methods in current use are not able to capture this; more sophisticated psychometric methods, such as the model-based approaches described above, may be better able to do so. Finally, it is possible that the lack of reliability is not an artefact or a methodological problem, but rather reflects a true lack of invariance in procedural learning; that is, that there are no stable individual differences to measure in this cognitive system.

Taken together, the results of the current meta-analysis demonstrate that procedural learning in the SRTT exhibits suboptimal test-retest reliability, irrespective of the sampling and methodological manipulations explored here. Split-half reliability, on the other hand, is considerably better, indicating some degree of consistency within sessions. While some design features contributed to small improvements in reliability, none resulted in adequate levels of test–retest reliability. While it is possible in principle that their cumulative impact could lead to significant increases in reliability, it was not possible for us to test this directly, because there is not yet sufficient data available in the field. Unfortunately, due to the lack of reporting of psychometric properties of the SRTT, further research is needed to adequately determine the impact of methodological factors by systematically investigating their influence on reliability. While it may not pose a major concern for group comparisons, individual differences research needs to be considered in light of the low measurement reliability of the SRTT [90]. The absence of correlations between measures thought to tap the same construct is often interpreted as pointing towards domain specificity or lack of shared variance between measures, when it may simply reflect attenuation due to measurement error [1]. Until adequate reliability is established for existing procedural memory tasks, or new reliable measures are developed, the field of procedural memory will continue to be hampered by underspecification of its components and a poor understanding of its relationship with cognitive constructs of interest, such as language.

## Data Availability

The dataset and scripts are available at https://osf.io/a65hn/).

## Footnotes

1 Buffington *et al.* [61] only reported a corrected estimate of reliability using Krus & Helmstadter [66]. This was done because the observed reliability was negative. Removal of this study from the analysis did not affect the overall estimate of split-half reliability (z = 0.81, *r* = 0.67).
